# Climate and soil effects on tree species diversity and aboveground carbon patterns in semi-arid tree savannas

**DOI:** 10.1038/s41598-023-38225-3

**Published:** 2023-07-17

**Authors:** Sylvanus Mensah, Florent Noulèkoun, Kangbéni Dimobe, Thomas Seifert, Romain Glèlè Kakaï

**Affiliations:** 1 Laboratoire de Biomathématiques et d’Estimations Forestières, Faculté des Sciences Agronomiques, Université d’Abomey Calavi, Cotonou, Benin; 2 Chair of Forest Growth, Albert-Ludwigs-Universität Freiburg, Freiburg im Breisgau, Germany; 3 Department of Environmental Science and Ecological Engineering, Korea University, 145 Anamro, Seongbukgu, Seoul, 02841 Korea; 4 Institut des Sciences de l’Environnement et du Développement Rural, Université de Dédougou, BP 176, Dédougou, Burkina Faso; 5 Department of Forest and Wood Science, Stellenbosch University, Matieland, 7602 South Africa

**Keywords:** Ecology, Forestry

## Abstract

Climatic and edaphic effects are increasingly being discussed in the context of biodiversity-ecosystem functioning. Here we use data from West African semi-arid tree savannas and contrasting climatic conditions (lower *vs*. higher mean annual precipitation-MAP and mean annual temperature-MAT) to (1) determine how climate modulates the effects of species richness on aboveground carbon (AGC); (2) explore how species richness and AGC relate with soil variables in these contrasting climatic conditions; and (3) assess how climate and soil influence directly, and/or indirectly AGC through species richness and stand structural attributes such as tree density and size variation. We find that greater species richness is generally associated with higher AGC, but more strongly in areas with higher MAP, which also have greater stem density**.** There is a climate-related influence of soils on AGC, which decreases from lower to higher MAP conditions. Variance partitioning analyses and structural equation modelling show that, across all sites, MAP, relative to soils, has smaller effect on AGC, mediated by stand structural attributes whereas soil texture and fertility explain 14% of variations in AGC and influence AGC directly and indirectly via species richness and stand structural attributes. Our results highlight coordinated effects of climate and soils on AGC, which operated primarily via the mediation role of species diversity and stand structures.

## Introduction

Uncovering the ecological mechanisms that underpin the effects of biodiversity on ecosystem functions across biomes and biogeographical regions is fundamental to inform global efforts aiming to achieve a win–win scenario of biodiversity conservation and climate change mitigation. Tree biomass and carbon storage are often studied in forests, as proxies for primary production and ecosystem functions, given their importance for ecosystem functioning and the global carbon cycle^1,2^. Empirical evidence suggests that plant diversity is positively linked with tree biomass and carbon storage in a range of terrestrial ecosystems^3–5^. However, this finding has been repeatedly challenged by studies reporting diverse patterns of species diversity-biomass carbon or productivity relationships across vegetation types and regions^4,6–9^. The inconsistency of the findings suggests that the relationships between species diversity and biomass or carbon storage are controlled by mechanisms (i.e., complementarity, selection and facilitation) operating under the interplay of complex abiotic factors including climatic, topographic and edaphic, which ultimately vary between scales, biogeographic areas and ecosystems^4,10^. This is also true especially in disturbed ecosystems in semi-arid environments, where periodic fires can reduce both tree diversity, density and above-ground biomass in the short term^11,12^, thereby reducing competition and allowing coexistence of competitors^13,14^.

It is generally well acknowledged that tree diversity and productivity or biomass/carbon storage are shaped by environmental gradients in climate, although the relative effects of these factors are scale-dependent^15–18^. At regional and global scales, climatic factors are reportedly the most important drivers because species survival and growth are mainly related to their tolerance range for larger-scale gradients in mean annual precipitation (MAP) and temperature (MAT)^16,19^. Specifically, MAP enhances both species diversity and aboveground carbon (AGC) in forest ecosystems^3,5,20,21^, whereas MAT reportedly either favours or limits species diversity and aboveground biomass (AGB) or carbon^22–24^. Because variations in species richness and carbon stock are associated with gradients in climatic factors, it should be expected that for a given scale, the effects of species diversity on AGC vary along environmental gradients.

Beyond climate, edaphic factors are also expected to play a fundamental role at the local scale, because local environmental conditions can act as a filter of plant community assembly and as a determinant of productivity due to gradients in resource (i.e., soil moisture and nutrients) availability^15,25^. Soil chemical properties such as pH, soil organic carbon (soc) that determine nutrient availability and soil texture including clay and silt content that regulate water availability, are reported as influential drivers of tree diversity and functioning^26,27^. Two hypotheses have been offered to explain the mechanisms underlying the effects of soil physical and chemical properties on community assembly: the soil fertility hypothesis and the inverse-texture hypothesis^28–30^. The soil fertility hypothesis states that high soil nutrient availability can promote niche differentiation and facilitation, which in turn, can lead to increased diversity, recruitment and growth; but it may also increase mortality and turnover rates through increased interspecific competition^31^. The inverse-texture hypothesis predicts that plant communities on coarse-textured (e.g., high sand or gravel content) soils should have higher above-ground net primary productivity than communities on fine-textured (e.g., high clay content) soils in arid and semi-arid ecosystems; the reverse trend is predicted to occur in humid regions^29,30^. Testing these hypotheses to understand how soil conditions influence species diversity and AGC in different climate conditions could provide new insights into how soils modulate the effects of climate on species diversity and carbon stocks.

The effects of climatic and edaphic factors on plant community assembly in semi-arid environments may not be mutually exclusive^11,18^, and previous studies have shown that the influence of tree species diversity on biomass production or productivity and their underlying mechanisms are controlled by the effects of these environmental factors^5,16,32^. Research studies that have investigated these aspects have also reported that the effects of diversity on forest productivity are more likely to be driven by biological interactions such as facilitation and complementarity in less productive or resources limited forest biomes^16,32^. Likewise, a more positive relationship between diversity and tree biomass has been reported in association with increasing environmental stress^33^, in line with the stress gradient hypothesis, which predicts that species interactions can shift from high competition in favorable environments to facilitation and lower competition in stressful environments^34–36^. However, it is not clear if this also holds true for semi-arid tree and shrub-dominated communities subject to continuous water scarcity. Furthermore, the influence of environmental conditions on AGC could also operate through stand structural attributes, which have consistently been reported as stronger drivers of AGC in forests^24,37^. Vegetation structures such as stand density and tree size variation are often related to tree biomass carbon and could provide additional insights on these effects on soil and climate on AGC and species richness.

In this study, we explored how the interrelated pathways between climate and soil variables shape the relationship between tree species richness and AGC storage, while also accounting for the potential mediation effects of stand structural attributes. We used data from 209 inventory plots in West African tree savannas, a largely underrepresented ecosystem in the current literature. In particular, we:

1. Assessed the bivariate relationships between species richness and AGC, and how climate modulates such a relationship. Because new species established in a community would likely contribute to the ecological processes and ecosystem functioning through facilitation and competition effects, we hypothesized that (1a) greater species diversity (species richness) would generally be associated with higher AGC. However, this effect could vary depending on climatic conditions, possibly due to species-specific growth response to climatic water-related stress. Specifically, because more humid conditions caused by enhanced climatic water and moisture availability prevailing in areas receiving higher precipitation can promote rapid natural regeneration and growth^38–40^, we hypothesized that (1b) species richness-AGC relationships would be substantially more positive in humid/warmer sites (i.e., higher MAP and MAT) than in dry/colder sites.
2. Assessed how species richness and AGC are influenced by soil variables such as pH, soil organic carbon and soil texture (sand, silt, and clay content) under contrasting climate (lower vs. higher MAP/MAT) conditions. Soil physico-chemical properties are important factors that alter local conditions for tree growth and survival^41–43^. In line with the inverse-texture hypothesis^29,30^, we predicted that (2) increasing fine particle content (e.g., silt and clay) in the soil would be associated with lower species richness and AGC across all sites and more strongly on sites with lower MAP.
3. Explored how climate and soil influence AGC directly, and indirectly through species richness and stand structural attributes such as stem density and tree size variation. Climate (e.g., MAP) and soil conditions can influence biomass production^44,45^ by impacting tree cover and vegetation growth. In this respect, we hypothesized that (3a) coordinated effects of climate and soil would determine AGC, with a main mediation role of species diversity and stand structural attributes. In line with previous studies^46,47^ which reported that both the stem density and tree size variation increased with species diversity (e.g., species richness), and that these stand structural attributes had a strong effect on forest tree biomass or carbon^5,48,49^, we predicted that (3b) the positive influence of species richness on AGC would manifest mainly via stand structural attributes.

## Material and methods

### Study area

**Figure 1:**
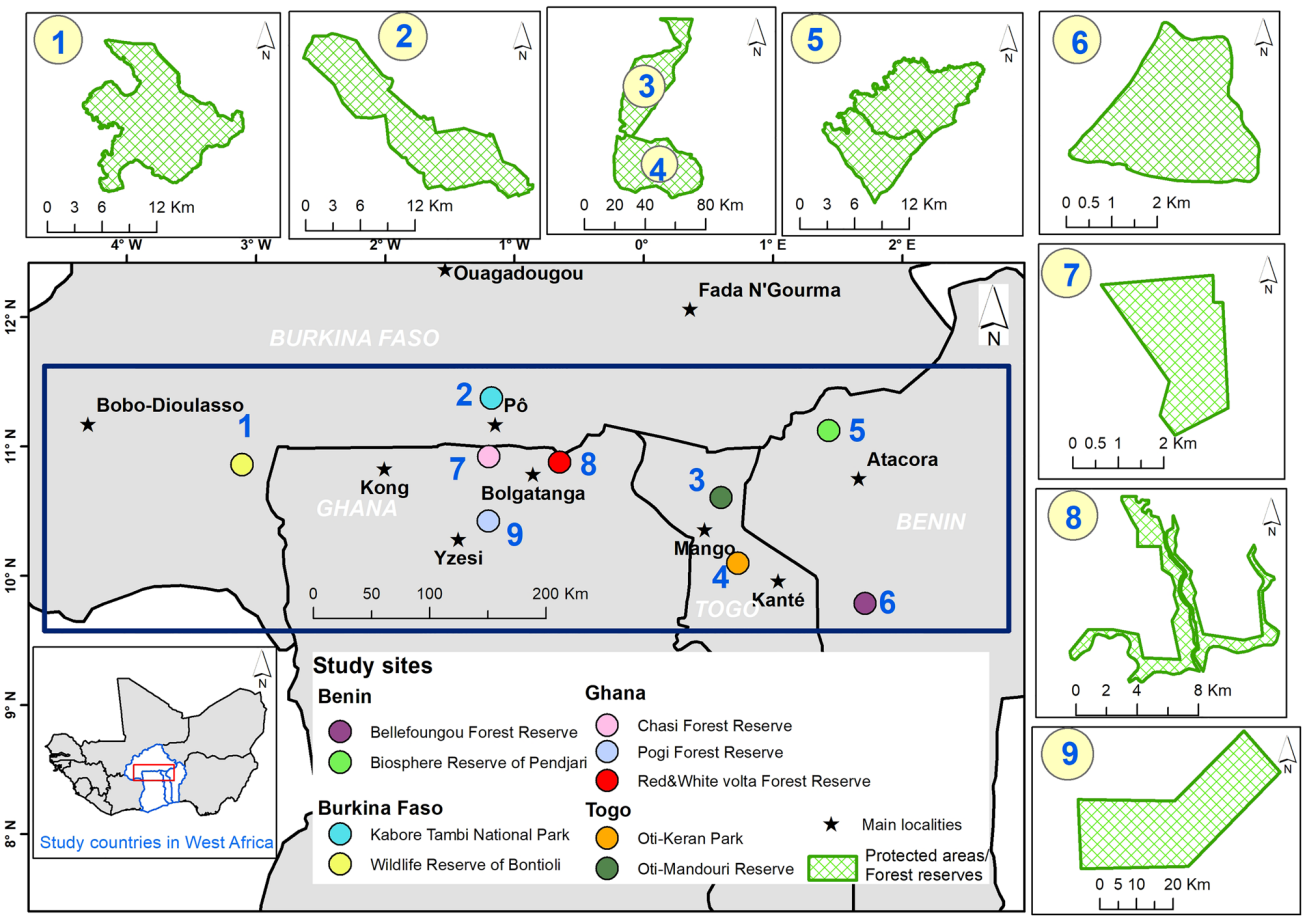
Map of the study area in West Africa, showing the locations of the sampling sites in Benin, Burkina Faso, Togo, and Ghana.

The study was carried out in West Africa (see map on Fig. 1, generated using QGIS version 3.28^50^; http://www.qgis.org). The study area covers parts of the semi-arid landscapes of Burkina Faso, Ghana, Togo and Benin. Phytogeographically, the study area is located in the West African Sudanian tree/shrub savannas and is characterized by a unimodal MAP distribution and high MAT. MAP ranges between 700 and 1300 mm. The rainfall lasts for about six months from May to October. The rainy season is followed by a long dry season between November and April. MAT varies between 24 and 32 °C. The main vegetation types in the study area are tree and shrub savannas with a grass layer dominated by annual grasses, as well as perennials.

We collected a wide spatial and extensive dataset of 209 plots of 0.1 ha each (50 m × 20 m), installed in the West African Sudanian tree/shrub savanna zones of the four countries (Benin, Burkina Faso, Togo and Ghana). The plots were established in the tree/shrub savanna zones encountered in the protected areas across the region following a systematic random sampling and standard guidelines established for inventories in semi-arid landscapes^51^ to cover a wide range of environmental variations. In total, 58, 23, 84 and 44 sampling points were installed in northern Benin, southern Burkina Faso, northern Togo and northern Ghana, respectively.

In northern Benin, sampling plots were established in the Biosphere Reserve of Pendjari and in the Bellefoungou Forest Reserve, located in the Sudanian climatic zone and the Sudano-Guinean transition zone, respectively of the Benin Republic. The Biosphere Reserve of Pendjari is ecologically interesting because it has not been managed for timber production and its spatial structure is largely the outcome of natural processes. The vegetation of the Biosphere Reserve of Pendjari is a mosaic of savannas, woodlands, dry forests and gallery forests along rivers. The MAP averages 1000 mm while the MAT is 27 °C. The Bellefoungou Forest Reserve is situated between 1°42′00′′ and 1°45′00′′ E longitude and 9°46′40′′ and 9°49′00′′ N latitude and is a complex of woodlands, gallery forests, savannas, and plantations^52^. The Bellefoungou Forest Reserve is characterized by the Sudano-Guinean transition zone climate of Benin^53^, with an average daily temperature of 28 °C, and MAP of 1200 mm.

In southern Burkina Faso, data were collected in two protected areas: the Total wildlife reserve of Bontioli, located in the Sudanian climatic zone of the country and the Kabore Tambi National park, located in the Sudano-Sahelian climatic zone. The main vegetation types in these two protected areas are characterized by a mosaic of tree/shrubs savannas and forests (woodland, dry forests and riparian forests). The climate is tropical with a unimodal rainfall regime that lasts for 4–6 months. In the Sudano-sahelian zone, MAP ranges between 600 and 900 mm, and in the Sudanian zone it varies between 900 and 1000 mm^54^.

In northern Togo, data were collected within Oti-Keran-Mandouri (OKM), a complex of two protected areas (Oti-Keran and Oti-Mandouri), covering about 179,000 ha with a core area of 41,914 ha. The complex lies within the flat plains of the Oti River basin and characterized by a semi-arid climate. The complex is close to the localities of Mango and Kante, which receive 1050 mm and 1200 mm of MAP, respectively, although average rainfall has decreased significantly in recent years^55^. Sudanian savannas, dry forests, and riparian forests constitute the vegetation types of this area^56^. The main vegetation type is tree savannas. Indurate tropical ferruginous soils and swampy tropical ferruginous soils are mainly distributed in this area^57^. The OKM complex is considered as a key conservation corridors of the savanna elephants in West Africa^56,57^.

In northern Ghana, data were collected in three forest reserves (Pogi, Chasi, Red & White volta) in the Upper East Region (UER), located in the northeastern part of Ghana, and cutting across two administrative boundaries—Bolgatanga municipality and Bongo district. The climate is also semi-arid, distinguished by the alternation of a distinct wet season from May to October and a dry season (November–April). The MAP is approximately 1000 mm. The soils in this region are predominantly shallow and characterized by limited availability of exchangeable cations and low levels of organic matter content^58^.

### Plot data

In each plot, the diameter at breast height (DBH) and total tree height were measured for all living trees with DBH ≥ 5 cm. The minimum DBH of 5 cm was used to include both trees and shrubs, which play important ecological functions in the region. The total tree height was measured from the base of the trunk to the tip of the tree using a clinometer. The DBH was measured with a diameter tape held tight and horizontal to the stem axis. In total, 4465 individuals from 127 species were measured in the 209 plots. Individual trees were identified at the species and genus levels following the flora of Benin^59^, Burkina Faso^60^ and Togo^61^. The “Taxonstand” package in the R statistical software, version 4.2.2^62^ was used to confirm the species names and crosscheck potential synonyms of species.

### Environmental variables

The environmental data tested in this study included two terrain variables (elevation and slope), two bioclimatic variables (MAP and MAT) and soil data. The terrain variables were extracted from the 30 m resolution Shuttle Radar Topographic Mission (SRTM) Digital Elevation Model (DEM). The bioclimatic data were obtained from the CHELSA database (https://www.chelsa.org) at a pixel resolution of 1 km^63^. The soil data were retrieved from the Africa Soil Profiles Database (https://www.isric.org) at a pixel resolution of 250 m. The soil parameters considered in this study included pH in H_2_O, soil organic carbon (soc) (g/kg), sand content (%), silt content (%), and clay content (%). In order to ensure integration with the DEM, the soil data and bioclimatic variables were resampled to a resolution of 30 m using the bilinear interpolation method. The terrain variables were computed using the System for Automated Geoscientific Analysis (SAGA) software. Thereafter, a matrix of predictors was developed by superimposing the inventory plots on the soil, terrain and bioclimatic spatial layers and extracting the corresponding values in R statistical software using the packages “sp”, “raster” and “rgdal”. A descriptive summary of the environmental variables can be found as Supplementary Table S1 online.

### Species diversity, AGC and stand structural attributes

We used the plot-level species richness, determined as the number of individual species in each plot, to quantify species diversity. We used species richness not only because it is the primary taxonomic diversity metric most often employed, but also because it has direct implications for biodiversity conservation especially in biomes where tree density is lower such as savannas.

To estimate the AGC, we first quantified the AGB for all individual trees present in the plots by applying the multispecies allometric biomass equation developed by Chave et al.^64^: AGB = 0.0673 × (ρ × DBH^2^ × H)^0.976^, where *ρ* is the species-specific wood density (g cm^−3^), DBH the diameter at breast height (cm), and H the total height (m). In the absence of regional biomass allometric models specific to West Africa, this equation can provide reliable estimates of AGB^7^. While tree DBH et height were obtained from the field inventory, data on species-specific wood density were extracted from local studies in West Africa^65–67^ and from the Global Wood Density Database^68^ when species (or genus) data were not available in the local studies. Then the AGB of each individual tree measured was summed up within each plot to obtain the plot-level AGB. The AGC stock was then calculated by applying a carbon fraction of 0.5^69–71^.

We used two measures of plot-level stand variables to characterize the stand structure: stand density and tree size variation. Stand density is the number of trees per plot reported to the hectare unit while tree size variation was quantified by the coefficient of variation of DBH (CoV-DBH) was calculated as the ratio in percentage (%) of the standard deviation to the mean of individual tree diameter:

$\mathrm{CoV}-\mathrm{DBH}=\frac{{s}_{k}}{{\overline{\mathrm{x}} }_{k}}\times 100$, where s_k_ is the standard deviation of all tree DBH within the kth plot expressed as:

${\text{s}}_{{\text{k}}} = \sqrt {\frac{{\sum \left( {{\text{x}}_{{\text{k}}} - {\overline{\text{x}}}_{{\text{k}}} } \right)^{2} }}{{n_{{\text{i}}} - 1}}}$; and ${\overline{\text{x}}}_{k}$ is the mean diameter of the kth plot expressed as: ${{ \overline{\text x}}}_{k} = \frac{{\mathop \sum \nolimits_{{{\text{i}} = 1}}^{n} {\text{x}}_{{\text{i}}} }}{{{\text{n}}_{{\text{i}}} }}$. The distributions of plot-level species richness, AGC, tree density and tree size variation are presented in the Supplementary Fig. S1 online.

### Statistical analyses

All statistical analyses were performed with the R statistical software. Prior to the main analyses, we investigated whether disturbances by fire were an important determinant of tree diversity and AGC. This was done because in the context of tropical tree savannas, seasonal fires act as component of ecological restoration^72^. Increasing fire frequency can induce disturbed ecosystems^14^ and may contribute in the short term to increased tree mortality^12^. We used the Terra and Aqua combined MCD64A1 Version 6.1 Burned Area data product to extract data on fire disturbance. The Terra and Aqua combined MCD64A1 Version 6.1 is a monthly, global gridded 500 m product containing per-pixel burned-area and quality information based on MODIS (see https://lpdaac.usgs.gov/products/mcd64a1v061/)^73^. The data on fire disturbance were quantified as the frequency of fire occurrence (i.e., the total number of times a pixel was classified as burnt) from 2001 to 2021. Out of the 209 plots, 62 were recorded as having actual frequencies data (from 0 to 20 fire events), while the remaining exhibiting large negative values were treated as missing data. Using the 62 plots with fire events, we tested the effects of fire frequency on plot-level species richness and AGC (see Supplementary Fig. S2 online). To better depict the patterns in our data, we also evaluated species richness and AGC relationship under two contrasting fire regimes (i.e. lower vs. higher fire frequency), which were identified using the 25th and 75th percentile scores of the fire frequency data. Plots with a fire frequency between 0 and 4 were classified as low fire frequency sites (29 plots) as opposed to fire hotspots classified as plots with a fire frequency ranging between 10 and 20 (15 plots) (Supplementary Fig. S2). The analyses of the plots with fire occurrence data showed a non-significant effect of fire frequency on species richness, and a marginal effect (p = 0.057) effect on AGC (Supplementary Fig. S2). Further, we found no significant species richness and AGC relationships under contrasting fire conditions (Supplementary Fig. S2).

Our first objective was to analyse the bivariate relationship between species diversity and AGC, and how MAP and MAT modulate the relationships between species richness and AGC. We first examined the species richness and AGC relationships across all sites (pooled data for all countries) and at country level, using scatter plots and testing the significance of the linear relationships. Because we were more interested in how climate modulates species richness and AGC relationship patterns, we used the 75th and 25th percentile scores of the MAP data across plots to separate plots with higher MAP (> 1119–1364 mm; 52 plots) from lower MAP (740–942 mm; 51 plots). Based on these two subsets, we assessed both the main and interaction effects of MAP and species richness on AGC, using a linear mixed-effects model with country as the random effect (Supplementary Table S2). Using the same approach, we also identified plots with higher MAT (> 28.4–28.9 °C; 50 plots) and lower MAT (24.9–27.5 °C; 49 plots) and tested for both main and interaction effects of MAT and species richness on AGC (Supplementary Table S2). The interaction effects in the mixed-effects models were represented graphically for better understanding of the magnitude and direction of the interaction.

Our second objective was to determine how species richness and AGC relate with soil variables under contrasting local climatic (i.e., lower vs. higher MAP/MAT) conditions. To do so, we used the same approach described above to test the significance of the effects of soil variables such as pH, soc, sand, silt, and clay content on AGC and species richness, separately under the predefined contrasting climatic conditions (Supplementary Table S3). All these variables were standardized to a mean of zero and unit variance to allow for equal weight so that their relative effects could be compared. The standardized slope coefficients of these mixed-effects models were plotted for better visualization and comparison. Please note that these results were presented only for MAP because MAT did not show significant interaction effects with species richness on AGC (Supplementary Table S2).

**Figure 2:**
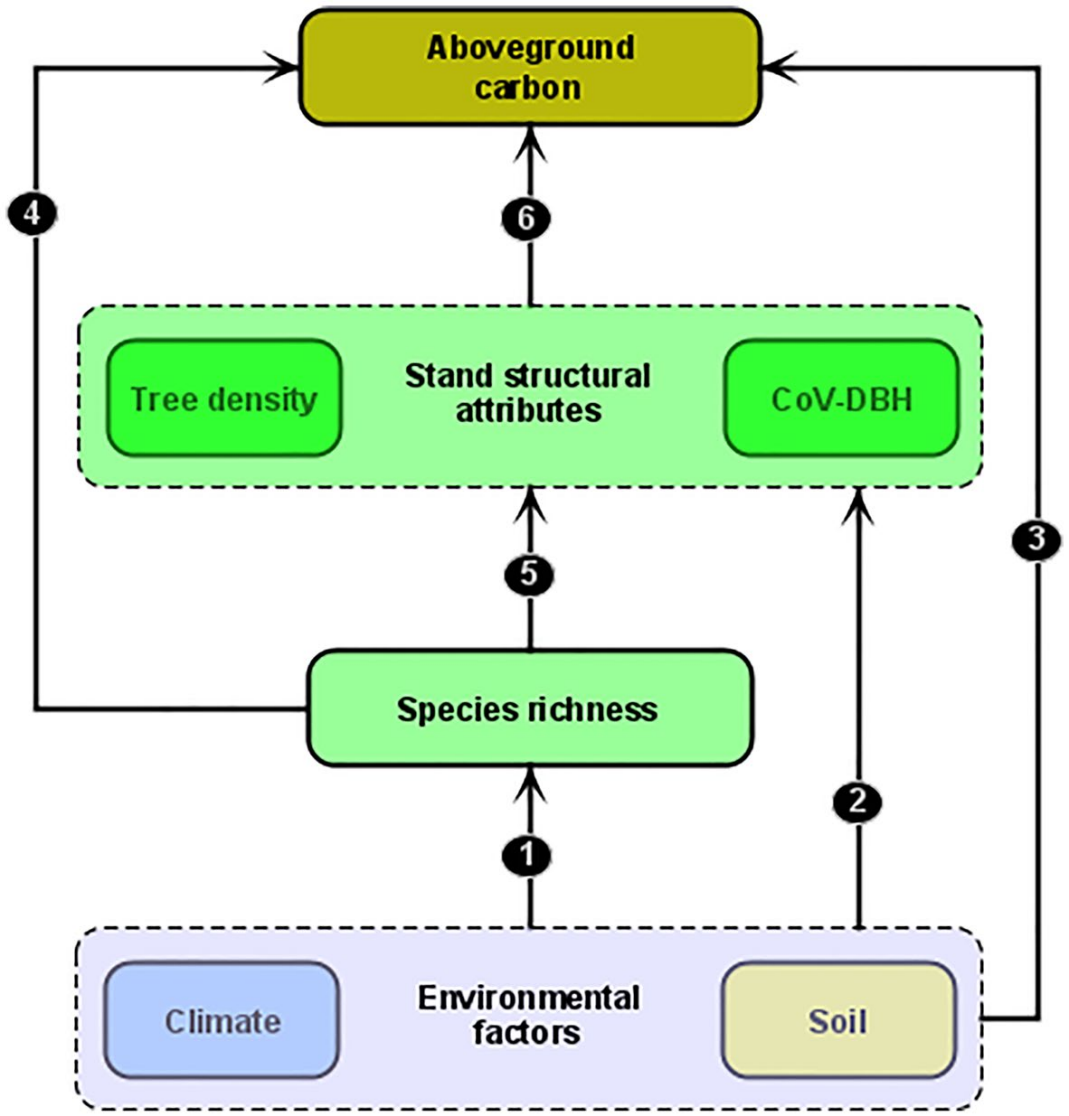
Conceptual model for testing how climate and soil influence AGC directly, and indirectly through species richness and stand structural attributes such as stem density and size variation. For description of the annotated paths (1–6) see “Statistical analyses” section.

In our third objective, we used structural equation modelling (SEM) to investigate how climate and soil variables influenced AGC directly, and indirectly through species richness and stand structural attributes such as stem density and tree size variation. To minimize redundancy or overfitting due to correlated soil variables, as shown in the Pearson-based correlation matrix heatmap (Supplementary Fig. S3), we summarized the soil variables information into two main dimensions using principal component analyses (PCA). After summarizing soil variables into two dimensions, other environmental variables such as slope, elevation, MAP and MAT could still be highly correlated with soil components or among themselves. Thus, to avoid multicollinearity in the analyses necessitating a simultaneous use of these variables, we tested for multicollinearity using the variance inflation factor (VIF) calculated based on mixed-effect models fitted with slope, elevation, MAT, MAP, the two soil PCA dimensions as predictors of species richness and AGC (Supplementary Table S4). We found that MAT and elevation exhibited VIF > 10 (Supplementary Table S4). In addition, MAT was also highly correlated with MAP (r = − 0.79). We therefore excluded both MAT and elevation and retained MAP and the two soil PCA dimensions for the SEM analyses. We built the SEM based on our conceptual model in Fig. 2. In line with the soil fertility and inverse-texture hypotheses, we predicted that increasing fine particle content (e.g., silt and clay) would reduce while nutrient-rich soils (e.g., high organic matter) would promote diversity (species richness; path 1), stand structural attributes (path 2) and AGC (path 3) (Fig. 2). We also expected that climate, especially increasing MAP would enhance climatic water and moisture availability which would promote tree density and species richness due to climate favorability (paths 1–3). Increasing species richness in benign environments would enhance AGC directly (path 4); but stand structural attributes such as stem density and tree size variation could also increase with species richness under the assumption of facilitating mechanisms of species coexistence and complementary use of resources (path 5) and would in turn strongly enhance AGC (path 6)^5,48,49^. Therefore, to test these hypotheses in the SEM, we first assessed how MAP and soil variables (i.e., the two PCA axes) co-determined species richness, stand structural attributes and AGC. Next, we evaluated the indirect relationships between AGC and these explanatory variables. In this respect, we tested for the direct influence of species richness on AGC and its indirect influence via stand structure, and how MAP and soil variables influenced AGC via species richness and stand structures. Finally, variance partitioning analysis was used to partition the variance shared by all explanatory variables used in the SEM. We estimated the unique and shared contribution of MAP, soil (PCA axes), species richness and stand structural attributes to AGC. The SEM was fitted using “lavaan’’ package^74^. The fits of the model to the data were evaluated by means of Chi square p-value, the goodness‐of‐fit index, the comparative fit index, and the standardized root mean square residual^75,76^. Variance partitioning analysis was performed using the ‘vegan’ package^77^.

## Results

### Bivariate relationships between species richness and AGC

**Figure 3:**
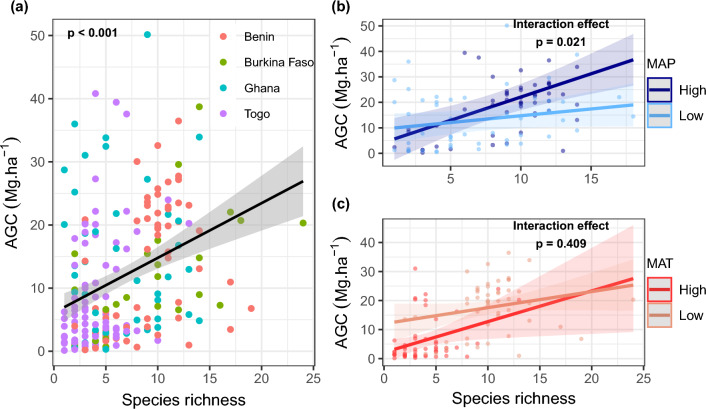
Relationships between species richness and aboveground carbon (AGC) across (**a**) all plots; (**b**) under lower and higher mean annual precipitation (MAP); and (**c**) under lower and higher mean annual temperature (MAT). Lower and higher MAP (resp. MAT) were determined using the 25th and the 75th percentile scores respectively, for the MAP (resp. MAT) data across the plots (see “Material and methods” section). The lines represent the fitted values and shaded regions the pointwise 95% confidence interval around the fitted values. See Supplementary Table S2 online for additional statistics on the interaction effects.

We found positive species richness and AGC relationships across studied sites in Benin (β = 0.41; p = 0.007), Burkina Faso (β = 0.38; p = 0.006) and Togo (β = 0.59; p < 0.001), but not in Ghana where a nonsignificant effect was noted (β = 0.02; p = 0.890) (Supplementary Fig. S4). Across all plots, AGC was positively related to species richness, which explained 12% of the overall variations in AGC (β = 0.36; p < 0.001; Fig. 3a). The linear mixed-effects models showed significant interaction effects of MAP and species richness on AGC (Fig. 3b; Supplementary Table S2). In this respect, increasing species richness increased AGC more strongly on sites with higher MAP than lower MAP (Fig. 3a). There was no significant interaction effect of MAT and species richness on AGC (Fig. 3b; Supplementary Table S2).

### Effects of soil variables on species richness and AGC in contrasting climate conditions

**Figure 4:**
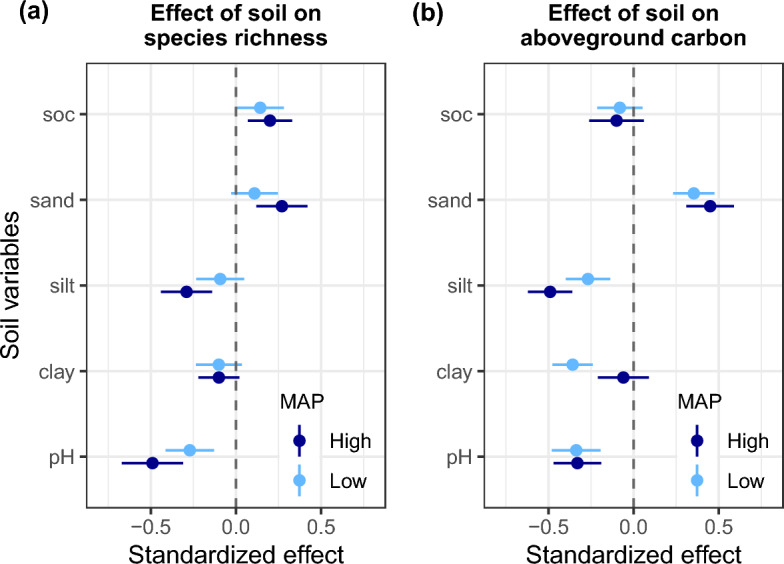
Effects of soil variables on species richness and aboveground carbon under contrasting climate conditions (low and high mean annual precipitation—MAP).

The effects of soil variables on species richness and AGC varied between the lower and higher MAP conditions (Fig. 4). Under lower MAP conditions, soc and all soil physical properties were not significantly associated with species richness, whereas in higher MAP conditions, increasing soc, sand and silt content tend to regulate species richness (Fig. 4a). For AGC, we found that the effects of clay content on AGC shifted from negative in low MAP conditions to neutral in high MAP areas (Fig. 4b). Overall, these results provided evidence for climate-related influence of soil properties on species richness and AGC.

### Direct and indirect effects of climate and soil variables on species richness, stand structural attributes and AGC

**Figure 5:**
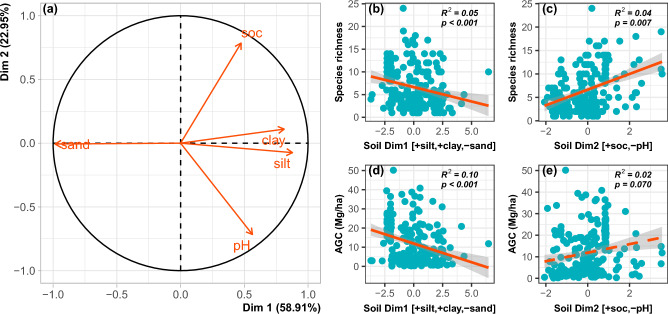
Relations between soil variables, principal components, species richness and AGC. In (**a**) we show the correlation of soil variables with the first two dimensions of the principal component analysis (PCA). In (**b**) we show the bivariate relationships of the two dimensions of the PCA with species richness and AGC.

Due to the number of candidate soil variables, we initially summarized the soil information into two main dimensions using PCA and retained the first two axes (Fig. 5a). The first principal component (Dim1), which captured 59% of the total variation, opposed sand content (negatively related to Dim1) to silt and clay contents (both positively related to Dim1). The second principal component (Dim2) accounted for 23% of the total information and opposed soc (positively associated with Dim2) to soil pH (Fig. 5a). As such, PCA Dim1 expressed a soil texture gradient from coarse- to fine-textured soils while Dim2 expressed a fertility gradient.

**Figure 6:**
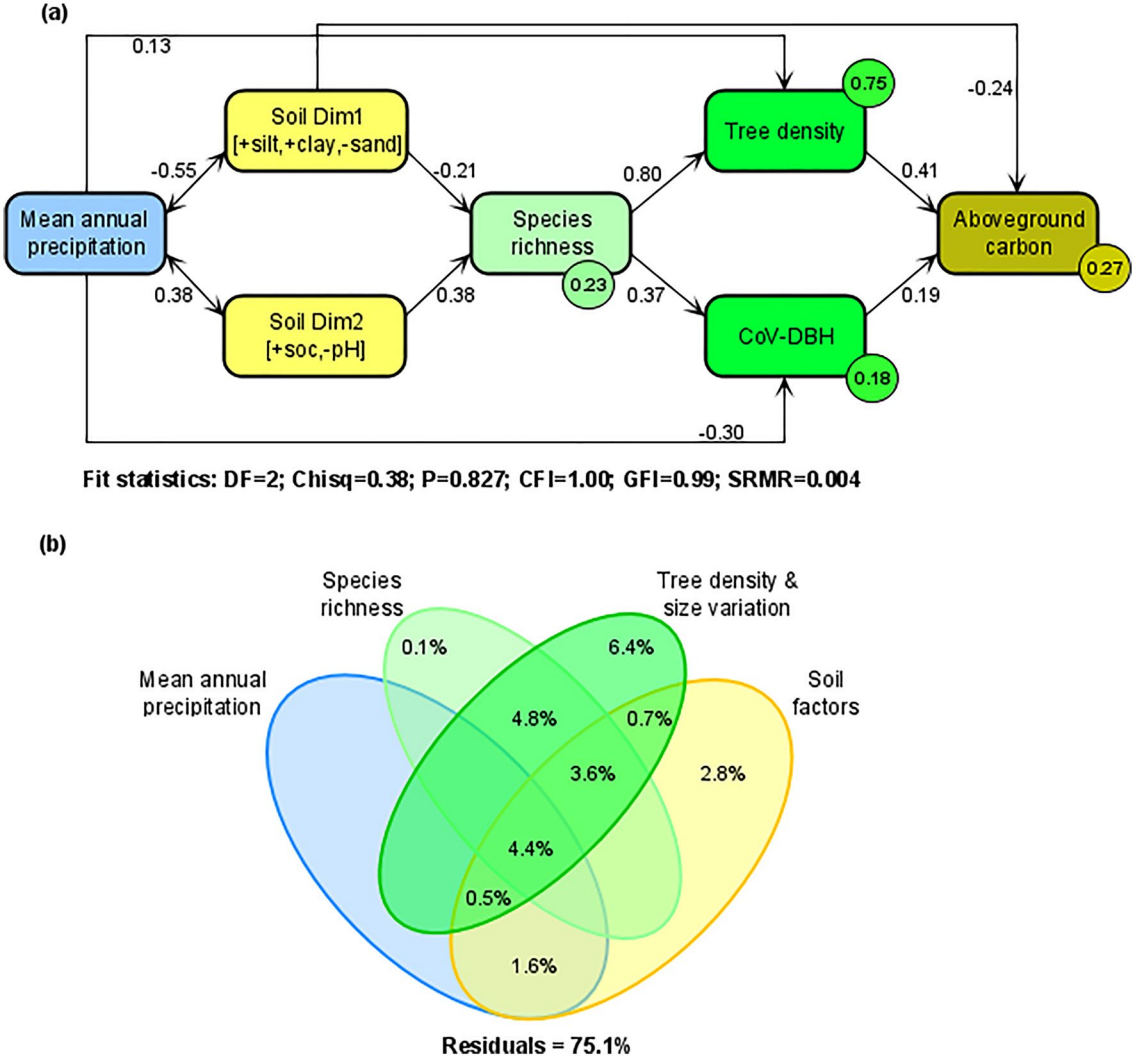
SEM paths (**a**) relating mean annual precipitation (MAP), soil texture (Dim 1) and soil fertility (Dim 2) to species richness, stand structural attributes (tree density and size variation—CoV-DBH) and aboveground carbon (AGC); and venn diagram (**b**) illustrating the shared and unique variance explained in AGC by MAP, soil variables, species richness and stand structural attributes. In (**a**), the single-pointed arrows represent causal paths, while the double-pointed arrows represent the correlations. The values on the arrows are the standardized path coefficients and their significance is presented in the Supplementary Table S5 online. Only significant paths (p < 0.05) were displayed for better readability. Coefficients of determination (R square) are shown in a circle next to each dependent variable. DF, degree of freedom; Chisq, Chi-square statistic; P, probability associated with Chi-square test; CFI, comparative fit index; GFI, goodness-of-fit index; SRMR, standardized root mean residual.

The SEM explained 27% of the variance in AGC and showed a good fit to the data, as evidenced by the acceptable range of the values of the comparative fit index, goodness of fit index and the standardized root mean residual (Fig. 6a). In terms of the direct effects, we observed that increasing species richness enhanced tree density and CoV-DBH, which in turn, promoted AGC. Increasing MAP increased tree density and decreased CoV-DBH (Fig. 6a). Both species richness and AGC were reduced in finer-textured soils, while species richness increased with an increasing gradient of soil fertility (Fig. 6a). These results are also confirmed by the bivariate analyses of species richness and AGC with the two soil PCA dimensions (Fig. 5b–d), although soil variables, relative to MAP, explained greater variance (14%) in AGC. In terms of indirect effects, our results showed that MAP influenced AGC indirectly through tree density and CoV-DBH but had a weak total positive influence (Supplementary Table S5). The negative association of soil Dim1 (soil finer-texture) with AGC was more strongly manifested through tree density than CoV-DBH (Supplementary Table S5). Meanwhile soil Dim2 (soil fertility axis) positive relation with AGC was mediated through CoV-DBH and tree density, with the latter exerting strong mediation effect (Supplementary Table S5). Finally, we noted that climate and soil factors were significantly correlated (Supplementary Table S5; Fig. 6a), suggesting that both climate and soils had coordinated effects on AGC, manifested through a central mediation role of species diversity and stand structural attributes. The variance partitioning analysis suggested that MAP, soil dimensions, species richness, and stand structural attributes explained 24.9% of the variance in AGC, with stand structural attributes and soil dimensions explaining greater variance than species richness and MAP (Fig. 6b).

## Discussion

We found mixed patterns of species richness and AGC relationships across the tree savannas in the four countries, suggesting that the evidence that species richness is a useful predictor of AGC is context dependent. In particular, we found a neutral pattern of species richness and AGC relationship on the Ghanaian sites. While this result runs contrary to the expectations of most biodiversity-ecosystem functioning (BEF) studies^3,78^, it supports the idea that increasing species richness does not always increase stand AGC^7,47^. Despite the fact that a considerable number of species was recorded in these stands (see Supplementary Fig. S1 online), they could still be more functionally redundant, possibly reducing opportunities for efficient use of space and resources through niche differentiation^5,47,48^.

While neutral and/or negative species diversity and AGC (or biomass/productivity) relationships have been previously reported, albeit fewer^4,9,79^, there is increasing empirical evidence from both experiments and natural systems that species richness and carbon relationships are generally skewed toward positive correlation patterns. Across the study sites (i.e., all data combined), our bivariate analyses suggest that species richness enhances AGC storage in West African semi-arid tree savannas (Fig. 3a). Recent global studies on diversity–biomass carbon (or productivity) relationships revealed a positive pattern at regional and global scale^3,44,78,80^. In this study, the positive effects of species richness on AGC are reminiscent of the findings in these global studies, which have already been explained by non-mutually exclusive mechanisms such as complementarity and selection effects where resource availability and environmental filtering play a central role^5,9,81–84^. As such, AGC can increase due to a few highly productive and dominant species (selection effects), or a better performance of coexisting species (e.g. shade vs. light demanding or deep vs. shallow rooted species) through increased resource use efficiency (niche complementarity).

In a recent study in West Africa, we showed that shifting diversity-carbon relationship at local scale can be explained by the effects of stand structural complexity and large-size tree^7^. However, at a larger spatial scale, environmental signals are expected to be larger and play a prominent role by influencing growth and ecosystem functioning^3,45^. Our results showed that both species richness and AGC were strongly related to climatic conditions. Particularly, we found that species richness-AGC relationships shifted from neutral in lower MAP areas to strongly positive in higher MAP sites (Fig. 3b). The shift in species richness-AGC relationships under contrasting climate conditions suggests that larger scale patterns of diversity and carbon are indeed regulated by climate, because species distributions, composition and growth performance are governed by climatic and tolerance range, and adaptations to physical conditions of the environment^85^. Our study was conducted in semi-arid tree savannas, where seasonal water availability would effectively promote plant growth and/or wood cover^13,40^. In this respect, the additional analyses (see Supplementary Fig. S5 online) showed higher stand tree density in higher MAP areas than lower MAP areas. Given the nonsignificant variation in mean diameter between lower and higher MAP plots (Supplementary Fig. S5), we assume that this shift in richness-AGC relationships could be a result of increased precipitation promoting more stems rather than larger tree size, through enhanced climatic water and moisture availability in higher MAP areas. Compared with lower MAP areas, the higher tree density in higher MAP areas may further imply more competition for soil resources, which would partly support the stress gradient hypothesis, although in the lower MAP areas, facilitation or reduced competition might not have been realized to the level that favors biomass production, due to limited hydrological niche breadths^86^. Overall, our findings indicate that the principal importance of climate for diversity and AGC stocks, as shown in other forest studies^3,78,80^, is also clearly evidenced in our semi-arid tree savannas.

To determine if the shifting species richness-AGC under contrasting climate conditions was also underpinned by the effects of soil factors, we analysed the climate-related effect of soil variables on species richness and AGC. We observed that both species richness and AGC responded independently to variation in soil factors, in line with the patterns observed in previous research^9,82^. The idiosyncratic patterns of the effects of soil factors on species richness and AGC partly challenge our understanding of how soil contributes to diversity effect on biomass. Nevertheless, we also found that the effects of soil variables on species richness and AGC varied between lower and higher MAP conditions (Fig. 4), providing evidence for climate-related influence of soil factors on diversity and carbon stocks. Additional analyses aiming to decompose the variance explained in AGC by MAP, soil variables, species richness and stand structural attributes in lower and higher MAP conditions revealed that stand structural attributes (tree density and size variation) and MAP explained more variation in higher MAP conditions than in lower MAP conditions, while the importance of soil variables decreased from lower to higher MAP sites (Supplementary Fig. S6). This sheds light on processes by which both soil physico-chemical variables and water-related stress co-determine variation in species diversity and tree carbon stock in semi-arid environments.

Soil attributes (soc, pH, sand, silt and clay content) are important factors of local conditions that affect plants. As such, these soil characteristics are also expected to influence carbon flux^82,87^. Sand silt and clay are properties of soil texture while soc is a key element that determines soil quality and fertility^88^. The bivariate analyses of species richness and AGC with the soil dimensions defined by the principal component analysis, i.e., texture and fertility gradients, showed that species richness and AGC were reduced in finer-textured soils, while species richness and AGC to a lower extent increased with the gradient of fertility. On the one hand, the negative association of finer-textured soils with species richness and AGC reinforces the importance of soil particles for tree growth, and particularly the detrimental effect of fine particles for tree carbon storage in water limited environment (Fig. 4b). Soils with more clay/silt content are likely to get more compacted in drier periods, thereby impeding on the potential of roots to penetrate or extend their network to access groundwater and soil nutrients^89^, although some plants can develop eco-physiological strategies to respond/cope with the stress. Linked to this, soils with relatively higher sand content are likely to facilitate the growth of roots to deeper soil layers for maximum uptake of groundwater in drier periods. Conversely, the effect of clay would be less detrimental for biomass production in high MAP areas as observed in Fig. 4b, due to enhanced soil water holding capacity. Hence, our result supports the inverse texture hypothesis for arid environments^29^. On the other hand, the positive relationship between soc (used here as an indicator for soil fertility) and diversity and AGC was expected, based on the soil fertility hypothesis^28^. However, adaptation of semi-arid tree species to local harsh climate and soil conditions can also promote retention of biomass and carbon, and some previous studies have argued that such adaptations can operate through the development of conservative resource-use strategies such as slow growth or high wood density, which potentially favor the accumulation of biomass at the stand level^3,81^. Overall, the significant effects of soil variables on both species diversity and AGC concur with past research that reported the effects of the environment on diversity and AGC stock in other ecosystems^45,87,90^.

Previous studies have shown that climatic variables such as MAP as well as soil variables drive tree biomass and carbon stock^3,47^. Other studies have further demonstrated that climate relative to soils can explain greater variations of growth and biomass patterns at large environmental scales^44,91^. Although there is evidence that MAP gradient modulates diversity-carbon stock relationships especially in the predefined contrasting climate conditions, it has a smaller influence than soils on AGC across all sites. Environmental drivers of AGC depend on context and spatial scales, and climate effects would ultimately be strong at larger spatial scales^45^. By extending our understanding of climate and soil effects on AGC with a focus on their interrelationships, our analyses (Supplementary Fig. S6) point out that climate and soils influence on spatial variation of carbon stock are not independent, and interactively varied across contrasting climatic conditions (Supplementary Fig. S6). Our SEM results revealed how interrelated pathways among multiple climate and soil factors shape tree diversity and aboveground carbon stock in tropical semi-arid savanna. More precisely, the SEM indicated that climatic and soil properties are correlated, and both influenced AGC through mediation of stand structural attributes and species richness. For instance, MAP regulates AGC via its positive effect on tree density and negative effect on tree size variation, as also observed in semi-arid broadleaf forests in China^33^. In addition, both soil texture and fertility regulated AGC via their influence on species richness and stand structural attributes (Supplementary Table S5). Together with the results of the variance partitioning analyses (Supplementary Fig. S6; Fig. 6b), these findings collectively suggest that climate and soil exerted coordinated effects on AGC, which manifested through the main mediation roles of species diversity and stand structural attributes. In this study, such coordinated effects are possibly underpinned by water and nutrient availability.

## Supplementary Information

Supplementary Information.

## Data Availability

The datasets analysed during the current study are available from the corresponding author on reasonable request.
