# Supplementary material for: Climate and soil effects on tree species diversity and aboveground carbon patterns in semi-arid tree savannas
Source: Sci Rep. 2023 Jul 17;13:11509. doi: 10.1038/s41598-023-38225-3 (PMC10352281; doi:10.1038/s41598-023-38225-3)
Supplement: Supplementary file 1 — Supplementary Information. [file 41598_2023_38225_MOESM1_ESM.pdf]

Climate and soil effects on tree species diversity and aboveground carbon patterns in semi-arid tree savannas

Sylvanus Mensah[1,2,*], Florent Noulekoun[3], Kangbeni Dimobe[4], Thomas Seifert[2,5] and Romain Glèlè Kakaï[1]

[1]Laboratoire de Biomathématiques et d'Estimations Forestières, Faculté des Sciences Agronomiques, Université d'Abomey Calavi, Cotonou, Benin

[2]Chair of Forest Growth, Albert-Ludwigs-Universität Freiburg, Freiburg im Breisgau, Germany

[3]Department of Environmental Science and Ecological Engineering, Korea University, 145 Anamro, Seongbukgu, Seoul 02841, Korea

[4]Institut des Sciences de l'Environnement et du Développement Rural, Université de Dédougou, BP 176, Dédougou, Burkina Faso

[5] Department of forest and Wood Science, Stellenbosch University, 7602 Matieland South Africa

*** Corresponding author**

Supporting Information

Supplementary Figure S1

Variations of plot-level species richness, aboveground carbon, tree density and coefficient of variation of tree diameter (CoV-DBH) across savanna sites in Benin, Burkina Faso, Togo and Ghana

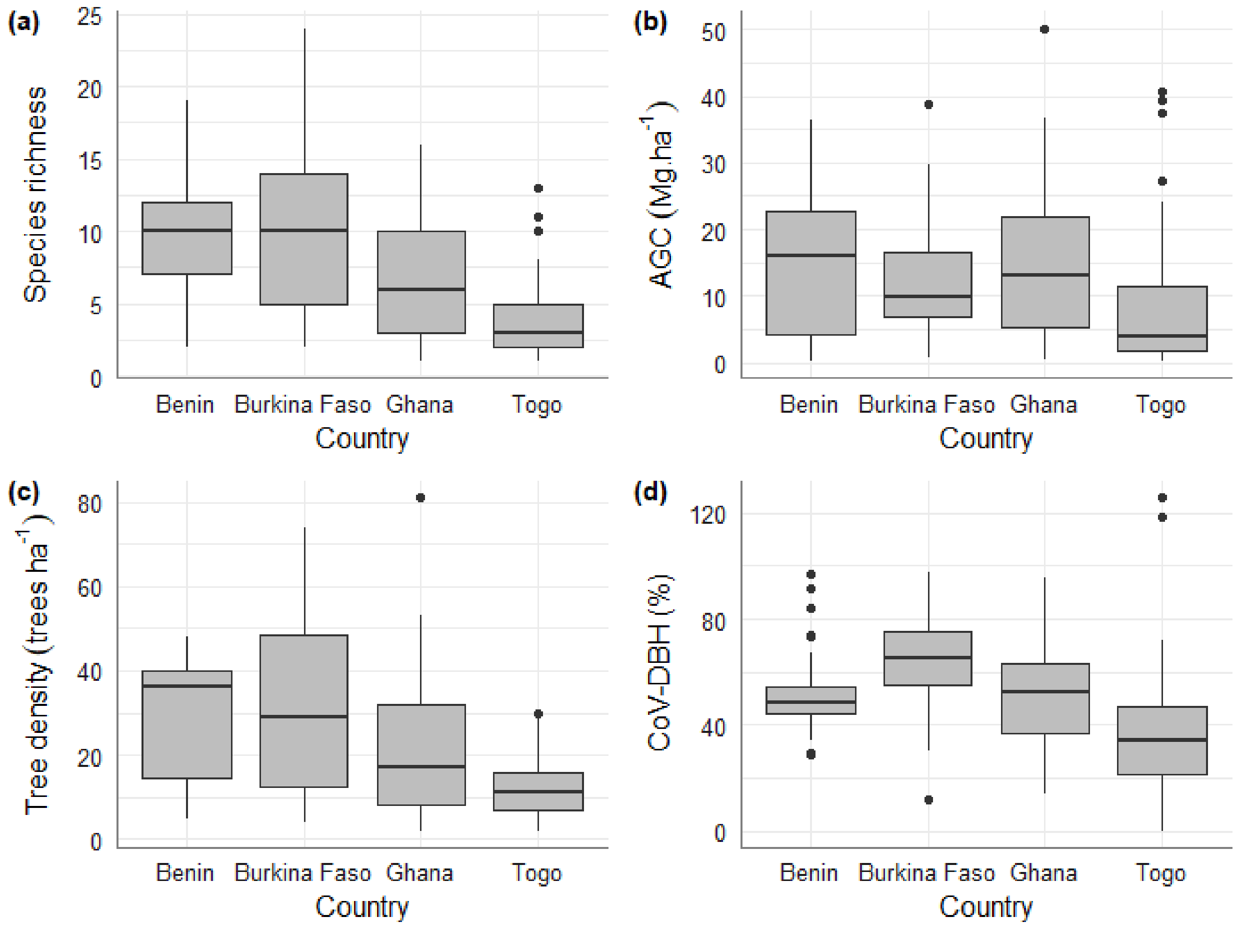

Supplementary Figure S2

Effects of fire frequency on species richness and aboveground carbon (AGC). Scatter plots are based on 66 sample plots with available fire data. The shaded regions represent the pointwise 95% confidence interval around the fitted values

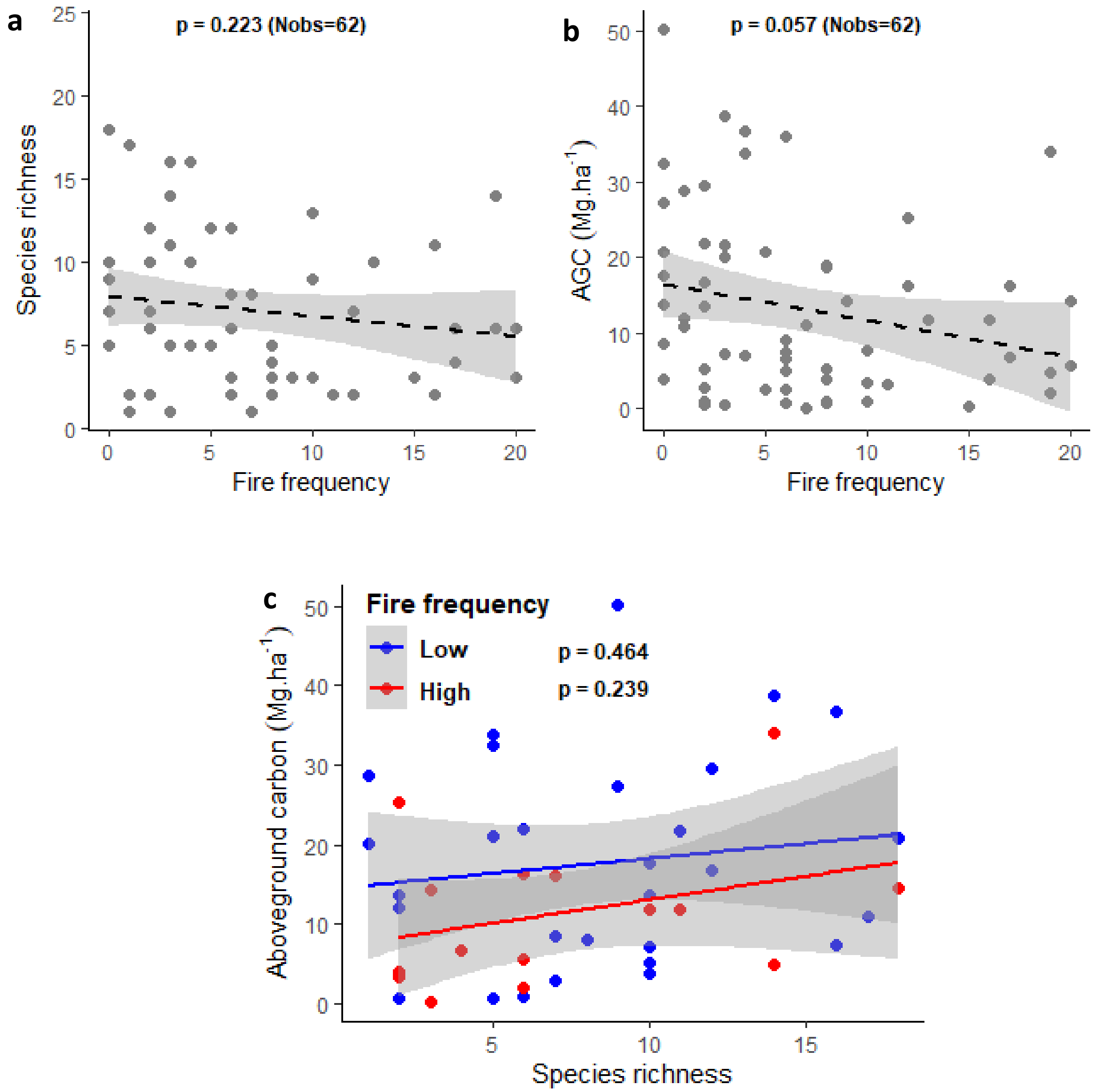

Supplementary Figure S3

Pearson correlations between soil physico-chemical properties used in this study

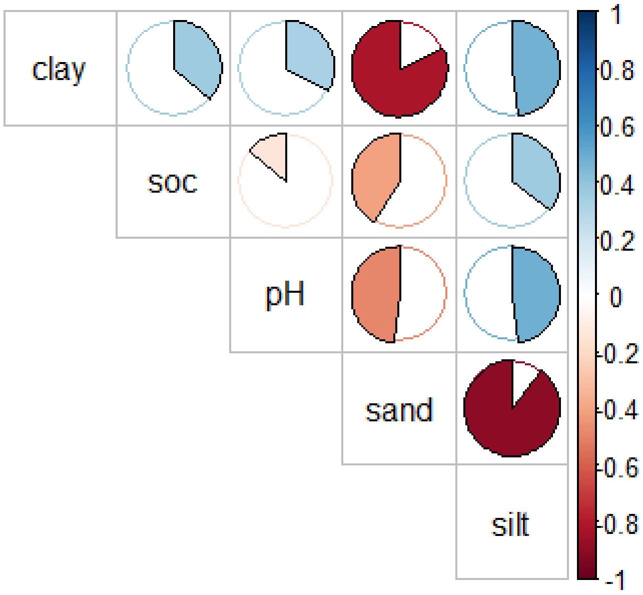

Supplementary Figure S4

Relationships between species richness and aboveground carbon (AGC) in tree savannas across the sampled areas. Point color other than gray shows the pattern of each country within the whole dataset. The colored lines are the fitted values while the shaded regions represent the pointwise 95% confidence interval around the fitted values

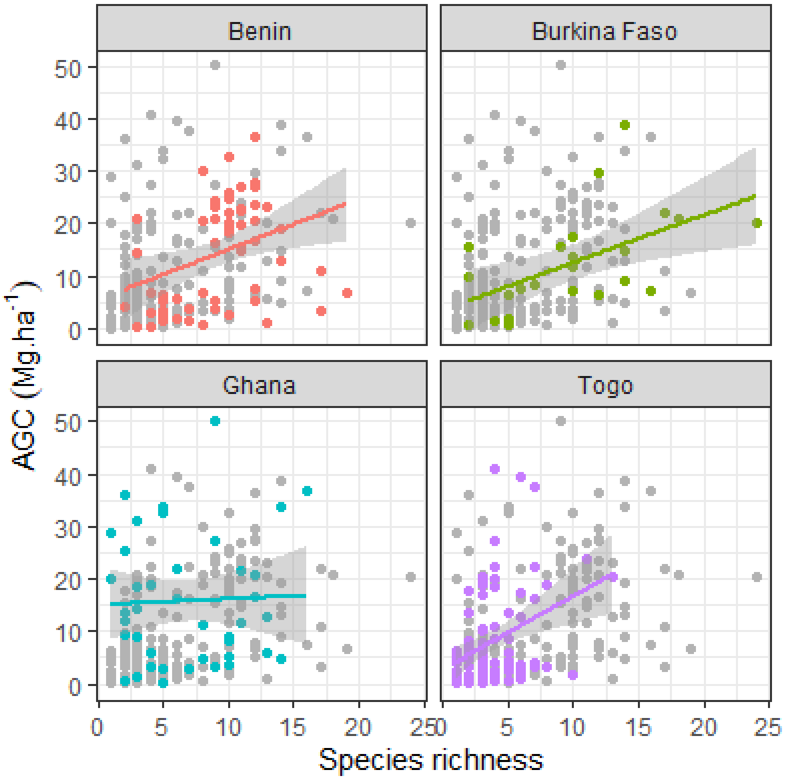

Supplementary Figure S5

Boxplots showing the distribution of plot-level tree density and mean diameter for low and high mean annual precipitation (MAP) sites

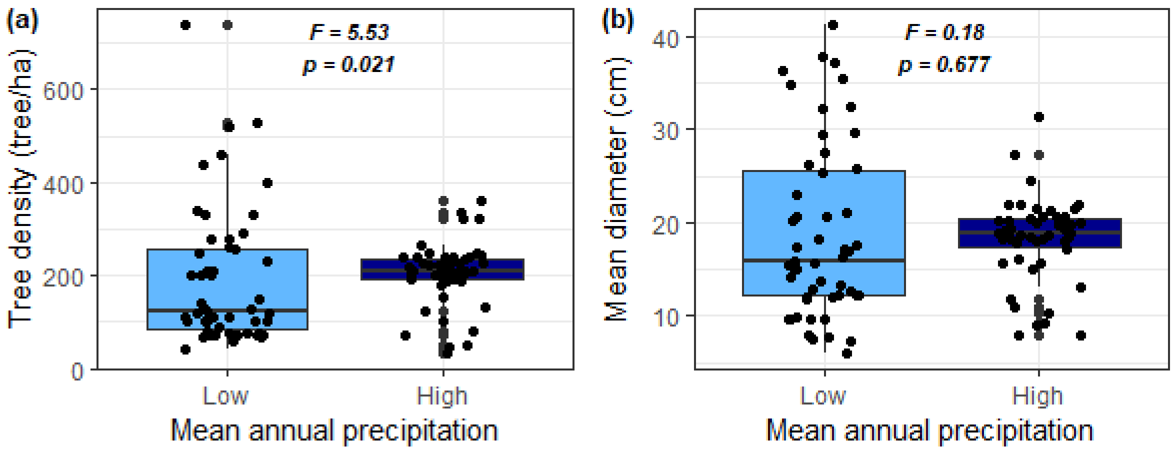

Supplementary Figure S6

Venn diagrams illustrating the shared and unique variances of aboveground carbon (AGC) explained by mean annual precipitation (MAP), soil variables (soil organic carbon, soc; pH; sand, silt and clay content), species richness and stand structural attributes (tree density and size variation) in (a) lower and (b) higher MAP conditions

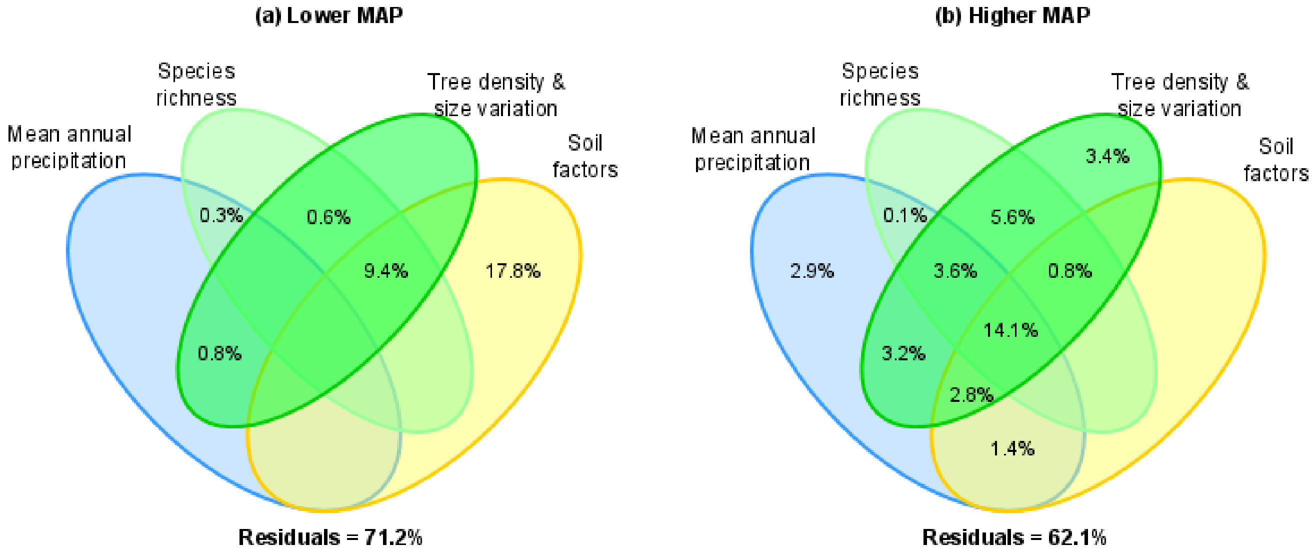

Supplementary Table S1

Descriptive statistics of environmental variables tested in this study.

Variables	Mean	Minimum	Maximum
Mean annual precipitation (MAP, mm)	1039	740	1364
Mean annual temperature (MAT, ◦C)	27.75	24.60	28.90
Elevation (masl)	246	119	641
Slope (%)	1.37	0.11	10.37
Soil organic carbon (soc, g/kg)	6.43	3.72	15.74
pH	6.2	5.6	6.8
Sand content (%)	49.3	30	64.4
Silt content (%)	22.8	16.6	32.7
Clay content (%)	27.4	17.9	36.9

Supplementary Table S2

Linear mixed-effects models testing for main and interaction effects of species richness and climate on aboveground carbon (AGC). Legend: numDF = Degree of freedom of the numerator, denDF = Degree of freedom of the denominator, F = Fisher statistics; MAP = Mean annual precipitation; MAT = Mean annual temperature

	NumDF	DenDF	F value	Pr(>F)
**Model: AGC ~ f (Species richness x MAP)**				
Species richness	1	95	16.007	<0.001
MAP	1	94.5	1.338	0.250
Species richness: MAP	1	93.2	5.551	0.021
**Model: AGC ~ f (Species richness x MAT)**				
Species richness	1	94	7.266	0.008
MAT	1	94	3.662	0.059
Species richness: MAT	1	94	0.687	0.409

Supplementary Table S3

Mixed-effect models testing for individual effects of soil variables (soil organic carbon, soc; pH; sand, silt and clay content) on species richness (SR) and aboveground carbon (AGC) in lower and higher MAP (mean annual precipitation) conditions. These effects were plotted in Figure 4. Std. Error = standard error

		Estimate	Std. Error	t value	Pr(>|t|)		Estimate	Std. Error	t value	Pr(>|t|)
Response variable	**Lower MAP**					**Higher MAP**				
SR	(Intercept)	-0.023	0.233	-0.098	0.929	(Intercept)	-0.414	0.446	-0.929	0.444
	soc	0.143	0.139	1.028	0.309	soc	0.192	0.122	1.579	0.121
	(Intercept)	-0.008	0.160	-0.050	0.968	(Intercept)	-0.065	0.411	-0.157	0.890
	pH	-0.271	0.142	-1.904	0.079	pH	-0.467	0.169	-2.760	0.010
	(Intercept)	-0.023	0.249	-0.091	0.934	(Intercept)	-0.261	0.464	-0.562	0.627
	sand	0.109	0.137	0.798	0.429	sand	0.257	0.137	1.880	0.066
	(Intercept)	-0.023	0.256	-0.089	0.935	(Intercept)	-0.237	0.440	-0.539	0.640
	silt	-0.092	0.141	-0.653	0.517	silt	-0.276	0.143	-1.934	0.059
	(Intercept)	-0.024	0.243	-0.097	0.929	(Intercept)	-0.399	0.496	-0.805	0.500
	clay	-0.100	0.135	-0.744	0.460	clay	-0.098	0.118	-0.824	0.414
AGC	(Intercept)	-0.081	0.300	-0.269	0.805	(Intercept)	-0.133	0.249	-0.535	0.653
	soc	-0.081	0.133	-0.605	0.548	soc	-0.093	0.149	-0.625	0.535
	(Intercept)	-0.063	0.246	-0.258	0.813	(Intercept)	0.000	0.136	0.000	1.000
	pH	-0.337	0.143	-2.351	0.023	pH	-0.317	0.137	-2.317	0.025
	(Intercept)	-0.063	0.251	-0.250	0.818	(Intercept)	0.000	0.130	0.000	1.000
	sand	0.354	0.122	2.911	0.005	sand	0.420	0.131	3.210	0.002
	(Intercept)	-0.063	0.261	-0.242	0.824	(Intercept)	0.000	0.126	0.000	1.000
	silt	-0.268	0.130	-2.055	0.045	silt	-0.466	0.128	-3.647	0.001
	(Intercept)	-0.069	0.257	-0.268	0.806	(Intercept)	-0.111	0.231	-0.481	0.687
	clay	-0.358	0.119	-3.020	0.004	clay	-0.062	0.145	-0.428	0.671

Supplementary Table S4

Mixed-effect models assessing the combined effects of climate, topography and soil variables on species richness and aboveground carbon (AGC), as separate response variables. The estimated variance inflation factors (vif) reveal high multicollinearity between temperature and elevation.

	Estimate	se	t value	Pr(>|t|)	vif
Species richness					
(Intercept)	1.884	0.118	16.023	0.000	
Precipitation	0.160	0.074	2.168	0.030	3.278
Temperature	0.452	0.179	2.520	0.012	**15.501**
Elevation	0.456	0.156	2.923	0.003	**8.583**
Slope	-0.063	0.040	-1.568	0.117	1.153
soilDim1[+silt, +clay, -sand]	-0.092	0.032	-2.926	0.003	2.047
soilDim2[+soc, -pH]	0.097	0.054	1.806	0.071	1.954
Aboveground carbon					
(Intercept)	0.062	0.157	0.394	0.722	
Precipitation	-0.089	0.132	-0.674	0.501	3.287
Temperature	-0.433	0.318	-1.359	0.176	**15.586**
Elevation	-0.041	0.248	-0.167	0.868	**7.625**
Slope	-0.081	0.069	-1.172	0.243	1.142
soilDim1[+silt, +clay, -sand]	-0.103	0.055	-1.854	0.065	2.081
soilDim2[+soc, -pH]	0.019	0.094	0.204	0.839	1.986

Supplementary Table S5

Significance of the SEM paths relating mean annual precipitation (MAP), soil Dim 1 (soil texture) and soil Dim 2 (soil fertility) to species richness, stand structural attributes (tree density and size variation – CoV-DBH) and aboveground carbon (AGC). A summary of these paths is provided in Figure 6a. Legend: Est.std: standardized estimates; se: standard error

		Est.std	se	z	p-value
Direct effects					
Path from	to				
MAP	Species richness	0.064	0.082	0.776	0.438
soilDim1	Species richness	-0.212	0.075	-2.835	0.005
soilDim2	Species richness	0.383	0.065	5.91	0.000
MAP	CoV-DBH	-0.308	0.083	-3.719	0.000
soilDim1	CoV-DBH	-0.088	0.08	-1.102	0.271
soilDim2	CoV-DBH	0.109	0.076	1.443	0.149
Species richness	CoV-DBH	0.377	0.068	5.523	0.000
MAP	Stand density	0.128	0.047	2.709	0.007
soilDim1	Stand density	-0.013	0.044	-0.303	0.762
soilDim2	Stand density	0.017	0.042	0.414	0.679
Species richness	Stand density	0.803	0.029	27.569	0.000
MAP	AGC	-0.003	0.084	-0.037	0.971
soilDim1	AGC	-0.235	0.075	-3.149	0.002
soilDim2	AGC	0.071	0.072	0.978	0.328
Species richness	AGC	-0.143	0.12	-1.198	0.231
CoV-DBH	AGC	0.191	0.065	2.934	0.003
Stand density	AGC	0.413	0.117	3.538	0.000
Correlations					
soilDim1	MAP	-0.546	0.045	-12.081	0.000
soilDim2	MAP	0.379	0.05	7.584	0.000
Indirect and total effects					
Indirect effect of species richness on AGC through stand density		0.332	0.095	3.486	0.000

Indirect effect of species richness on AGC through CoV-DBH	0.072	0.028	2.57	0.010
Sub-total indirect effect of species richness on AGC	0.404	0.098	4.105	0.000
Total effect of species richness on AGC	0.260	0.07	3.745	0.000
Indirect effect of soil Dim1 on AGC via species richness and stand density	-0.071	0.032	-2.198	0.028
Indirect effect of soil Dim1 on AGC via species richness and CoV-DBH	-0.015	0.008	-1.903	0.057
Sub-total indirect effect of soil Dim1 on AGC	-0.086	0.037	-2.331	0.020
Total effect of soil Dim1 on AGC	-0.321	0.081	-3.944	0.000
Indirect effect of soil Dim2 on AGC via species richness and stand density	0.127	0.043	2.964	0.003
Indirect effect of soil Dim2 on AGC via species richness and CoV-DBH	0.028	0.012	2.338	0.019
Sub-total indirect effect of soil Dim2 on AGC	0.155	0.047	3.317	0.001
Total effect of soil Dim2 on AGC	0.225	0.084	2.688	0.007
Indirect effect of MAP on AGC through stand density	0.053	0.024	2.156	0.031
Indirect effect of MAP on AGC through CoV-DBH	-0.059	0.026	-2.283	0.022
Indirect effect of MAP on AGC through species richness	-0.009	0.014	-0.651	0.515
Sub-total indirect effect of MAP on AGC	0.017	0.022	0.759	0.448
Total effect of MAP on AGC	0.013	0.087	0.156	0.876
